# Optimization of self-catalyzed InAs Nanowires on flexible graphite for photovoltaic infrared photodetectors

**DOI:** 10.1038/srep46110

**Published:** 2017-04-10

**Authors:** Ezekiel A. Anyebe, I. Sandall, Z. M. Jin, Ana M. Sanchez, Mohana K. Rajpalke, Timothy D. Veal, Y. C. Cao, H. D. Li, R. Harvey, Q. D. Zhuang

**Affiliations:** 1 Physics Department, Lancaster University, Lancaster LA1 4YB, UK; 2 Department of Electrical Engineering and Electronics, University of Liverpool, Liverpool L69 7ZF, UK; 3 Department of Physics, University of Warwick, Coventry CV4 7AL, UK; 4 Stephenson Institute for Renewable Energy, University of Liverpool, Liverpool L69 7ZF, UK; 5 Key Laboratory of Optoelectronic Chemical Materials and Devices, Jianghan University, Wuhan 430056, China; 6 Institute of Fundamental and Frontier Sciences, University of Electronic Science and Technology of China, Chengdu 610054, China

## Abstract

The recent discovery of flexible graphene monolayers has triggered extensive research interest for the development of III-V/graphene functional hybrid heterostructures. In order to fully exploit their enormous potential in device applications, it is essential to optimize epitaxial growth for the precise control of nanowire geometry and density. Herein, we present a comprehensive growth study of InAs nanowires on graphitic substrates by molecular beam epitaxy. Vertically well-aligned and thin InAs nanowires with high yield were obtained in a narrow growth temperature window of 420–450 °C within a restricted domain of growth rate and V/III flux ratio. The graphitic substrates enable high nanowire growth rates, which is favourable for cost-effective device fabrication. A relatively low density of defects was observed. We have also demonstrated InAs-NWs/graphite heterojunction devices exhibiting rectifying behaviour. Room temperature photovoltaic response with a cut-off wavelength of 3.4 μm was demonstrated. This elucidates a promising route towards the monolithic integration of InAs nanowires with graphite for flexible and functional hybrid devices.

Semiconductor nanowires (NWs) have emerged as potentially important building blocks for future photonic and electronic devices due to their unique properties including superior light absorption, enhanced photo-carrier separation and epitaxial growth insensitive to lattice mismatch^1^. Particularly, InAs NWs are highly promising for applications in high-speed electronics and mid-infrared optoelectronic devices owing to their narrow direct band gap (0.35 eV), small electron effective mass and high electron mobility (~30,000 cm^2^/Vs at 300 K)^2^,^3^,^4^. Graphene/graphitic films on the other hand exhibit excellent electrical and thermal conductivity, high mechanical robustness, superb transparency, mechanical flexibility and unique optical properties^5^ which has made them an ideal transparent conducting electrode to replace conventional indium tin oxide (ITO)^6^,^7^. The monolithic integration of semiconductor NWs with graphene/graphitic substrates (GS) has stimulated enormous research interest over recent years as it would enable the exploitation of these exceptional material properties. Together with the scalability^8^,^9^ and relative aboundance of graphene, this technology provides a unique platform for the development of high performance, flexible and cost-effective optoelectronic nanodevices, such as flexible displays, printable electronics, sensors, light emitting diodes and flexible solar cells^10^,^11^,^12^. Several devices have recently been demonstrated including hybrid graphene/ZnO NWs^11^ and graphene/n-Si Schottky junction^13^ solar cells and light emitting diodes^14^.

In contrast to conventional semiconductor substrates which possess covalent bonding, GS lacks surface dangling bonds which makes them chemically inert to foreign atoms. As a result NWs growth on GS is very challenging^5^. The small bond length of the graphene lattice^15^ (1.4 Å) compared to that of III-V materials presents another major obstacle to overcome. Unlike the growth of II-VI NWs such as ZnO on graphene^10^,^16^,^17^,^18^ and graphitic substrates^19^ which has been extensively investigated with optimized conditions, the growth of III-V semiconductor NWs on GS is currently in its formative stage and requires extensive studies for growth optimization^5^. However, compared to the traditional III-V substrates, graphite (like all other carbon related materials) is highly conductive thermally^20^,^21^ and temperature-sensitive^22^,^23^. These features imply a significant modification to the adatom kinetics during epitaxy in particular, a high rate of re-evaporation^24^ which would result in a low yield of NWs ensembles. Moreover, it has been demonstrated^10^ that the surface morphology and density of ZnO nanostructures on graphene are strongly temperature dependent. Although InAs NWs are more sensitive to growth conditions when compared to other III-V materials^25^, the growth parameter study and the control of InAs NWs morphology and distribution on GS is still lacking. It is well established that the size and distribution of NWs can be controllably manipulated using basic growth parameters^26^,^27^. Hence, an investigation of the influence of growth parameters and the conditions for realizing optimal InAs NWs with controlled geometry is crucial. Furthermore, most III-V semiconductor NWs are plagued by the presence of wurtzite (WZ) and zinc-blende (ZB) crystal phase mixtures (polytypism)^28^,^29^,^30^ which function as traps for carriers,^31^ reduce carrier mobility^32^ and conductivity^33^. Polytypism also results in electron scattering at stacking faults or twin planes^34^,^35^ which is detrimental to their optical properties and device applications. As a result, to exploit the potential of NWs/GS, it is crucial to investigate the conditions for realizing InAs NWs/graphite structures with controllable geometry and low defect density. The few available reports of self-catalyzed^36^,^37^,^38^ and Au-assisted^15^ InAs NWs growth by metal-organic chemical vapor deposition (MOCVD) and GaAs^39^ NWs growth by molecular beam epitaxy (MBE) on GS have been limited to basic NWs synthesis. Additionally, the yield of vertically-aligned InAs NWs is relatively low ~[(2–7) × 10^8^ cm^−2^] and requires more extensive studies for growth optimization^5^. In a previous study, we reported the growth of vertically aligned InAs NWs on graphite with highly uniform diameters through optimized In droplets^28^. The growth of high quality, nontapered and ultrahigh aspect ratio InAs_1−x_Sb_x_ NWs was also demonstrated on graphite^29^. It was shown that NW nucleation and growth is facilitated by the predeposited In droplets. The In-assisted growth of InAs and InAsSb NWs on Si substrates was also demonstrated^40^,^41^,^42^. In this paper, we seek to demonstrate the control of morphology and spatial distribution of InAs NWs as a function of growth parameters including growth temperature (T_G_), growth rate (ξ) and V/III flux ratio (Ψ) with the aim of growth optimization. In particular, we intend to obtain a high yield of vertically-aligned InAs NWs with high aspect ratio and superior crystal structure for functional device applications. It is demonstrated that the self-catalyzed growth of InAs NWs with significantly improved morphology can be achieved within a relatively narrow temperature window and restricted domain of growth rate and V/III flux ratio. In comparison to previous reports of self catalyzed InAs NWs, a significantly reduced density of defects was observed in as-grown nanowires.

## Results and Discussion

### Optimization of InAs Nanowire Growth

**Figure 1:**
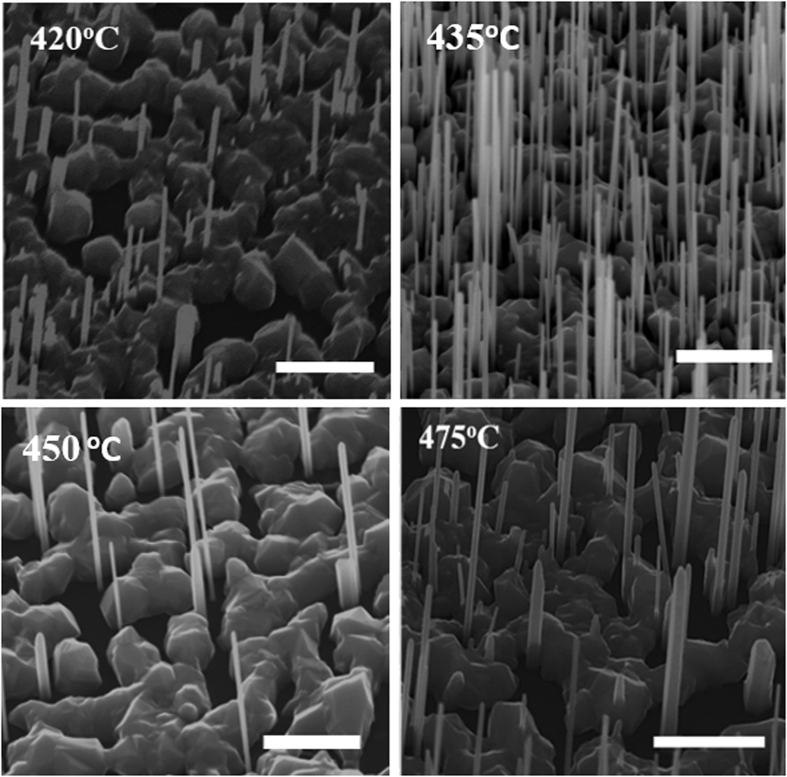
45° tilted SEM images of InAs nanowires grown on graphitic substrates with a fixed In-flux at various temperatures in the range of 400–475 °C. The scale bars correspond to 1 μm.

**Figure 2:**
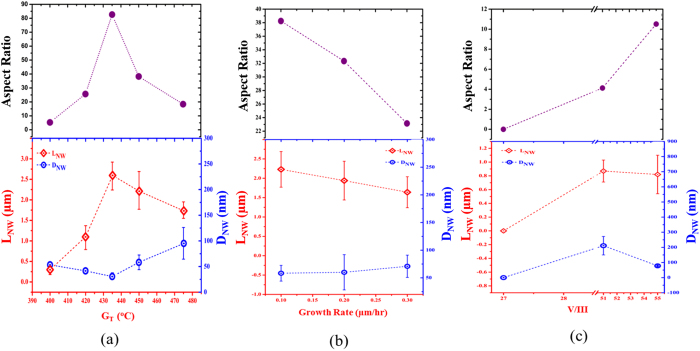
Aspect ratio (top), length (L_NW_) and diameter (D_NW_) (bottom) of InAs nanowires grown on graphitic substrate as a function of varied growth temperature (T_G_) (**a**); growth rate (**b**) and V/III flux ratio (**c**).

The scanning electron microscope (SEM) images in Fig. 1 shows the dependence of InAs NWs density and morphology on growth temperature (T_G_) in sample series A. The NWs exhibit a homogeneous diameter along their entire length without any measurable tapering. At the lowest temperature (400 °C), a dominant cluster (islands) growth was observed. A slight increase in T_G_ to 420 °C yielded a sparse NW distribution (∼5.28 × 10^8^ cm^−2^), and a maximum yield of ∼8.09 × 10^8^ cm^−2^ was obtained for a further increase in temperature to 435 °C (Fig. 1b). Increasing T_G_ into the 450–475 °C range led to a decrease in NW density as shown in Fig. 1(c,d). The observed dependence of NW density on T_G_ can be explained by the kinetic modifications to the distribution of pre-deposited In droplets^30^. A kinetically inhibited adatom mobility at a low temperature (400 °C) promotes the development of small but highly dense droplets leading to the growth of surface clusters at the expense of NWs. An increase in T_G_ in the range of 420–435 °C results in the formation of slightly large but well seperated droplets which favour the growth of high density of NWs. It is therefore not surprising that the highest yield of vertically-aligned NWs was obtained at 435 °C. However, for higher temperatures (435 °C < T_G_ ≤ 475 °C), the nucleation droplets merge to form clusters and are consumed at the early stages of NW growth. In addition, the desorption rate of the droplets is significantly increased leading to less nulceation of NWs^43^. Figure 2(a) shows the variation of NWs length (L_NW_) as a function of T_G_. Over 70% of measurable NWs in each sample were analyzed for each point. Gaussian approximations were then used for the determination of the error bars of the NWs geometry (L_NW_ and D_NW_) which is expressed as the deviation from the mean geometry of normally distributed NWs. It can be seen that L_NW_ increases for a T_G_ increase in the range of 400–435 °C while it decreases in the temperature range of 435 °C < T_G_ ≤ 475 °C. Maximum L_NW_ was thus obtained at a T_G_ of 435 °C which is consistent with a previous report^44^. It is worth noting that the dependence of L_NW_ on T_G_ is accompanied by an inverse dependence of diameter (D_NW_) on T_G_, e.g. the D_NW_ decreases first (up to 435 °C) then increases with a further rise in T_G_. Consequently, long and thin NWs with a high aspect ratio (~83) was obtained at an optimal temperature of 435 °C (length and diameter of 2.58 ± 0.34 μm and ~31.21 ± 6.59 nm respectively). This also corresponds to the temperature which yields a high density of NWs. Notably, despite the high aspect ratio, the NWs remain vertically well-aligned without the presence of randomly oriented NWs. This demonstates the feasibility of fabricating InAs NWs with good geometry which would allow for fundamental studies such as size-dependent quantum confinement effects. The observed phenomenon can be interpreted in terms of the diffusion-limited growth of NWs in MBE. It has been theoretically and experimentally shown that the axial growth of NWs by MBE is strongly dependent on adatom diffusion from the substrate to the droplet^45^,^46^.

Consequently, at a low T_G_ of 400 °C, adatom diffusion is kinetically impeded and axial NW growth is suppressed in favour of cluster growth. A slight rise in T_G_ to 420 °C favours the diffusion of adatom towards the top of the NWs with considerable deposition on the sidewalls leading to the emergence of short and thick NWs. An increase in T_G_ to 430 °C further enhances the diffusion flux of adatoms with minimal deposition along the sidewalls. This translates to a surge in L_NW_ with a corresponding shrinkage in D_NW_^24^,^47^,^48^. However, for the upper temperature limit (T_G_ > 435 °C), adatom incorporation probability and diffusion flux is significantly reduced due to unfavourable chemical potential gradient^49^ and re-evaporation^24^. Generally, NW growth was realized on graphite within the temperature domain of 400–475 °C which falls within the MBE growth window of InAs NWs (400–500 °C) 2,44,50.

**Figure 3:**
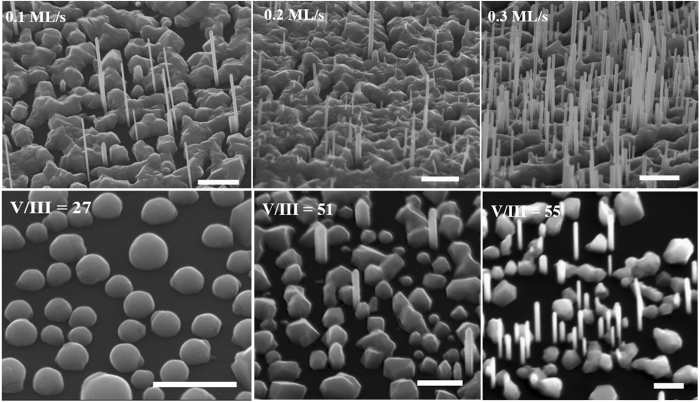
45° tilted SEM images of InAs NWs grown on graphite at a constant growth temperature and As-flux but different growth rates (top panel) and V/III flux ratios (bottom panel). The scale bars for varied growth rates and V/III flux ratio images corresponds to 1μm and 500 nm respectively.

Turning to sample series B, there was a clear and monotonic increase in NWs density with increasing growth rate (ξ) (Fig. 3, top panel). A high yield (6.44 × 10^8^ cm^−2^) of vertically-aligned NWs was obtained at a relatively high ξ of 0.3 ML/s. The increase in NW density with increasing ξ is associated with the dependence of NWs nucleation on In-flux. Higher In-flux results in the formation of a high density of In droplets due to the reduction in adatom diffusion time^30^. This translates to an increase in NWs density as a function of ξ. This observation implies the availability of high NW growth rate which is favourable for realizing cost-effective devices. Intriguingly, the average L_NW_ slightly decreases with increasing ξ while D_NW_ remains nearly constant (Fig. 2b) leading to a slight decrease in NW aspect ratio with an increase in ξ. The observed decline in axial growth rate is understandable given the sharp rise in NWs density at constant supply of precursor flux which implies a reduction in the available growth species due to its consumption during NWs nucleation. In addition, the increase in ξ favours an increase in adatoms surface coverage with a suppression of adatom surface mobility. This results in a decreased diffusion flux of the adatoms from the substrate surface to the growth interface leading to a reduction in L_NW_.

The influence of V/III flux ratio (Ψ) on InAs NWs growth in sample series C is depicted in Fig. 3 (Bottom panel). It reveals that NW nucleation is strongly dependent on Ψ, whereas no NW growth was realized at a relatively low Ψ of 27, an increase in Ψ to 51 resulted in a few NWs (2.55 × 10^7^ cm^−2^). The NW nucleation probability was enhanced by utilizing a highly As-rich conditions (Ψ = 55) evidenced by the increase in NWs yield. This demonstrates that an As-rich condition (Ψ ≥ 51) is required for suppressing the growth of clusters in favour of vertically-aligned InAs NWs. The dependence of NW aspect ratio on Ψ in Fig. 2c also suggests that the axial NWs growth is promoted by As-rich conditions. Although there was no significant change in L_NW_ for a slight increase in Ψ to 55, it enabled the growth of thin NWs.

### Structural property of as grown Nanowires

**Figure 4:**
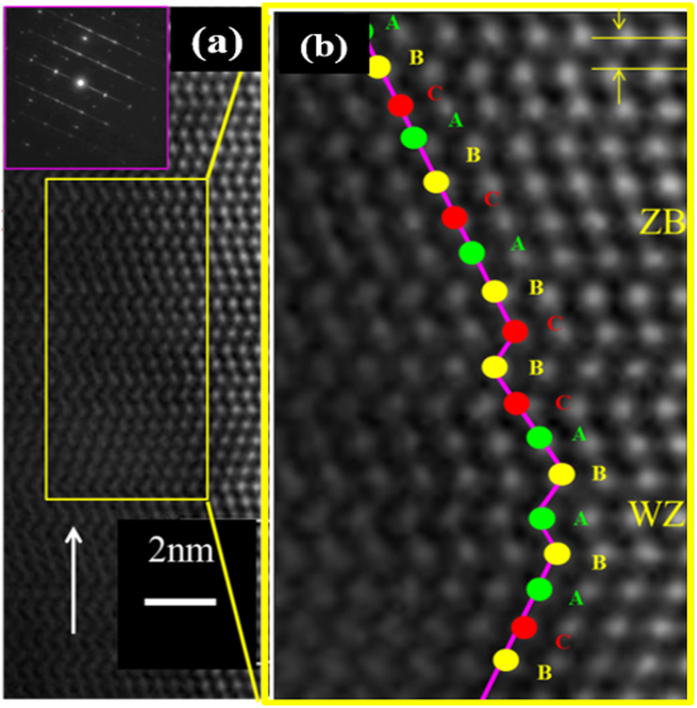
Typical HR-TEM image of InAs nanowires grown on graphite [the inset shows the selective area electron diffraction pattern] (**a**); an enlarged section of the HRTEM image (**b**).

To gain insight into the crystal structures of as-grown NWs, high resolution transmission electron microscope (HRTEM) experiments were performed. However, in order to quantify the distribution of defects present in the NWs, a clear distinction between stacking faults (SFs) and rotational twins (RTs) is made. A III-V semiconductor bilayer (BL) is composed of a pair of atomic layers with vertically stacked group III and group V atoms. The crystal structure of a semiconductor is dictated by the sequential arrangement of the BLs. A typical Zinc Blende (ZB) sequence is …ABCABC… while a Wurtzite (WZ) sequence is …ABABAB… where each letter represents a bilayer. A SF results from a partial distortion of the vertical stacking sequence either by the absence of a segment in the normal sequence or the inclusion of a single segment of the other crystal structure. In the ZB phase, a sequence of ABCABABC indicates a SF with the fault line between C and A leading to the inclusion of a WZ unit (AB) between the ZB segments. Similarly, for a WZ phase a SF exists in a stacking sequence of the form ABACBAB due to the inclusion of C which alters the regular WZ sequence. On the other hand a RT is created when a segment of a crystal is rotated by 60° around the growth axis (〈111〉) such that it is translated to a mirror image of the regular segment. The interface between the regular and mirror segments is referred to as the twin boundary. A stacking sequence of ABAB C BABA for WZ phase and ABC A CBA for the ZB phase illustrates the presence of RTs. A representative high resolution TEM micrograph (Fig. 4a) shows the NWs/GS exhibits a ZB/WZ crystal phase mixture consistent with previous reports of self-assisted InAs NWs^1^,^2^,^51^. An enlarged segment of the HRTEM image (Fig. 4b) clearly demonstrates the transition between the ZB and WZ phases with SF present. This polytype behaviour of III−V NWs has become the rule rather than the exception^35^,^52^,^53^ and has been mostly associated with the lower surface energy of the WZ phase in comparison to the corresponding crystalline orientation of the ZB material. As a consequence the WZ phase is more stable in NW structures with high surface to volume ratio^27^,^54^. Classical nucleation theory has also been used to explain this phenomenon and attributed it to the lower WZ nucleation barrier compared to that of ZB^55^,^56^,^57^. The electron diffraction pattern (inset of Fig. 4(a)) further confirms the mixed ZB/WZ crystal structure with multiple SFs on the (111)/(0001) plane evidenced by the observable streaks in the diffraction pattern. An interplanar d-spacing of ~0.35 nm along the growth direction was obtained for the ZB segment (Fig. 4b) which corresponds to that of bulk ZB InAs (0.35 nm). Compared to our previous report^30^ of InAs NWs, a significantly lower density of defects (SFs and RTs) was observed in the InAs/GS samples. A typically InAs NWs/Graphite contains about 225.35 ± 56.34 SFs per μm. We attribute this improvement to the well optimized growth conditions. Similarly, no dislocations were observed in the NWs which can be associated with the van der Waals epitaxy (vdWE) growth technique. One of the key advantages of vdWE is the absence of interfacial lattice mismatch induced strain^58^,^59^ owing to the circumvention of lattice-matching requirements. In addition, there is a near coherent in-plane lattice matching (0.49%) between InAs and the graphene honeycomb lattice. The distance of 4.263 Å for the graphene honeycomb-honeycomb along 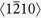 closely matches that of 4.284 Å for the nearest As-As or In-In bonds of ZB InAs^37^,^38^,^39^.

### Hybrid InAs/Graphite Infrared Photovoltaic Detectors

**Figure 5:**
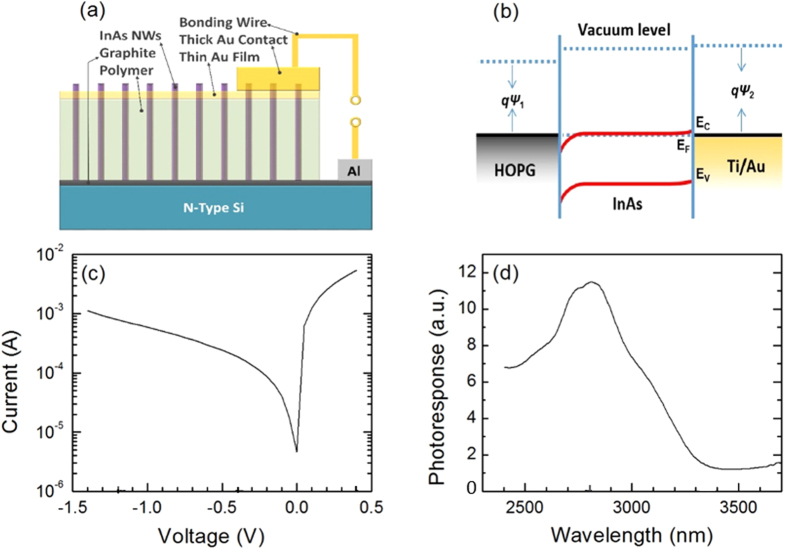
Schematic diagram of the NWs ensemble photodetector (**a**), bandgap diagram of InAs/Graphite heterojunction (**b**), I-V curve of a InAs NW ensemble/graphite hybrid device (**c**), and the room temperature spectral photoresponse of the hybrid device (**d**). The device mesa has a diameter of 25 μm.

To investigate the electrical properties of as-grown NWs, InAs/Graphite infrared photovoltaic detectors with various mesa diameters of 25–200 μm were fabricated (see Method for device processing). Each mesa contains InAs NW ensambles with an estimated number of over 3000 based on the areal density. The bandgap diagram of the device^60^,^61^,^62^ in thermal equilibrium is shown in Fig. 5(a). The work function of Graphite is around 4.3–4.7 eV^6^,^9^,^63^,^64^,^65^, while the Fermi level (*E*_F_) of InAs is ~5.0 eV^60^,^63^. This perspective leads to a downward band bending at the interface of InAs/Graphite which forms a depletion region for the holes. Such a depletion region would enable photodetection. A typical I-V profile of such device is shown in Fig. 5(b). It exhibits an asymmetric rectifying behaviour as expected from InAs/Graphite interface. The spectral photoresponse under the exiting wavelength of 2.4–3.7 μm is shown in Fig. 5(c). A photoresponse with a cut-off wavelength of 3.4 μm clearly implies photodetection from InAs NWs/graphite hybrid junction. The main peak located around 2.7 μm enables the device to work under part of near-infrared and mid-infrared bands.

## Conclusion

In conclusion, the optimal conditions for the MBE growth of InAs NWs on graphite is explicated. It has been discovered that the NWs geometry and areal density are sensitive to the growth parameters of temperature, growth rate and V/II flux ratio. The areal density and length of InAs NWs increases as the growth temperature is increased from 400 to 435 °C, a further increase induces a decrease in both, while a reversed dependence of diameter was observed within the same temperature regime. NWs on GS with high aspect ratio can be realized within a narrow temperature window of 420–450 °C. As-rich conditions were found to promote NWs growth. In addition, the GS favours high growth rate of InAs NWs which is promising from a device perspective. Our study also reveals that the NWs exhibit significantly reduced defect density which is attributable to the well optimized growth conditions. This study enables the controllable epitaxy of high quality InAs NWs on GS which paves the way for the integration of InAs NWs with graphite for the fabrication of advanced functional devices.

## Methods

### InAs Nanowire Synthesis on Graphite

The InAs NWs were grown on GS by solid-source MBE. Mechanically exfoliated graphite films from highly oriented pyrolytic graphite (HOPG) were transferred onto Si (111) substrates and subsequently loaded into the system and thermally outgassed. Indium (In) droplets were pre-deposited on the films prior to growth initiation at pre-optimised conditions (In-flux of 2.2 × 10^–7^ mbar and deposition temperature of 220 °C). The size of the droplet was about 70 nm with a number density of 6.33 × 10^8^ cm^–2^. Three series of samples (A–C) were grown as a function of T_G_, ξ and Ψ respectively. Firstly, in order to identify the effect of growth temperature, T_G_ was varied in the range of 400–475 °C at a fixed ξ and Ψ of ~55 for a growth duration of 60 min in sample series A. Then, the samples of series B were grown at various growth rates ranging from 0.1 to 0.3 ML/s at a fixed Ψ and constant T_G_ of 450 °C, to investigate the dependence of InAs NWs growth on ξ. Finally, the samples of series C were grown at a fixed T_G_ (450 °C) and In-flux with varying Ψ in the range of 27–55 to study the influence of Ψ on NW growth. For all the samples, the NWs growth was initiated and terminated by simultaneously introducing and shutting of both In and As supplies.

### Microscopy

The surface morphology of the NWs was investigated using a FEI XL30 SFEG scanning electron microscope (SEM). Transmission electron microscope (TEM) images were taken with a JEOL-JEM 2100 microscope working at 200 kV.

### Device Fabrication

The devices were processed using standard photolithography. A layer of SU8 with a thickness of around 4 μm was deposited via spin coating and UV exposure to encapsulate the nanowires. The SU8 was then thinned via reactive ion etching using a mixture of O_2_ and SF_6_ (35/5 sccm’s respectively) to free the tips of the nanowires. Circular devices with varying diameters (25–200 μm) were defined by a thin (~10 nm) gold layer which was deposited on and between the exposed tips of nanowires. To allow electrical connection to these devices a thicker top contact layer of Ti/Au (20/200 nm) was defined over some specific part of the device area for Ohmic contact. A back Ohmic contact of Aluminium with a thickness of about 200 nm was finally deposited on the reverse of the substrate to form a complete current loop.

### Device Testing

The electrical Current-Voltage (I-V) profile of the devices was measured with a Keithley Source Measure Unit. The spectral response was determined using phase sensitive detection comprising a lock-in-amplifier, and an iHR320 monochromator with a blaze grating wavelength of 2 μm was used in conjunction with a globar light source.

## Additional Information

**How to cite this article**: Anyebe, E. A. *et al*. Optimization of self-catalyzed InAs Nanowires on flexible graphite for photovoltaic infrared photodetectors. *Sci. Rep.* **7**, 46110; doi: 10.1038/srep46110 (2017).

**Publisher's note:** Springer Nature remains neutral with regard to jurisdictional claims in published maps and institutional affiliations.
